# Multirotor UAV—A Multidisciplinary Platform for Teaching Mechatronics Engineering

**DOI:** 10.3390/s25041007

**Published:** 2025-02-08

**Authors:** Denis Kotarski, Marko Pranjić, Ayham Alharbat, Petar Piljek, Toni Bjažić

**Affiliations:** 1 Department of Mechanical Engineering, Karlovac University of Applied Sciences, 47000 Karlovac, Croatia; marko.pranjic@vuka.hr; 2 Smart Mechatronics and Robotics Research Group, Saxion University of Applied Sciences, 7513 AB Enschede, The Netherlands; a.alharbat@saxion.nl; 3 Robotics and Mechatronics (RaM) Group, University of Twente, 7500 AE Enschede, The Netherlands; 4 Faculty of Mechanical Engineering and Naval Architecture, University of Zagreb, 10000 Zagreb, Croatia; petar.piljek@fsb.unizg.hr; 5 Department of Mechanical Engineering, Zagreb University of Applied Sciences, 10000 Zagreb, Croatia; toni.bjazic@tvz.hr

**Keywords:** Croatian Qualifications Framework, engineering education, multirotor UAV, PX4 ecosystem, rapid prototyping

## Abstract

This paper provides a comprehensive guide for educators on how multirotor UAV platforms can be utilized to achieve various learning outcomes in undergraduate mechatronics education. This study is based on a PX4 ecosystem combined with the MATLAB Simulink programming environment, covering both hardware and software aspects to support engineering education. The paper explains (i) which learning outcomes can be obtained, (ii) how mathematical models can be derived and implemented in simulation software, (iii) which hardware components are essential, their approximate costs, and possible upgrades based on available budgets, and (iv) which experiments students can perform using the UAV platform. A proposed educational prototype integrates airframe parts produced using additive manufacturing technologies with standard multirotor components. Additionally, a series of experiments were designed, including extensive testing of the multirotor control module. Three learning outcomes related to UAV hardware were incorporated into the engineering curriculum, while two software-related outcomes were addressed through student workshops. Future plans include the implementation of multiple UAV platforms in the educational process to further enhance learning outcomes.

## 1. Introduction

Due to the increasing applications of unmanned systems in various fields, there is a significant demand for engineers who are skilled in Unmanned Aerial Vehicle (UAV) system design, control, and maintenance, as well as mission planning and execution. A number of different UAV systems have been developed in recent years for numerous applications [1,2,3], ranging from conventional fixed-wing models to Vertical Take-Off and Landing (VTOL) UAVs. Generally, UAVs can be divided into fixed-wing aircraft, rotary-wing aircraft (or rotorcraft), hybrids, and flapping-wing aircraft. The rotary-wing aircraft can be further split into helicopters and multirotors. The main advantage of rotary-wing aircraft is their ability to perform a vertical and stationary flight and to move at moderate speeds, unlike fixed-wing aircraft, which require a runway or ramp for takeoff. Fixed-wing aircraft use aerodynamic speed to produce the necessary lift force using streamlined lifting surfaces. In contrast, rotary-wing aircraft rely on spinning propellers to produce lift force using their own power, which can be energy inefficient and impact flight endurance. Apart from having to lift its weight, rotary-wing must counteract the yawing moments by using the rotary blades in pairs. These drawbacks can be compensated for using hybrid UAVs, which have the ability to vertically take-off using rotor blades and then transition to horizontal flight using the fixed wings to generate lift force [4].

The rapid development of micro-controllers, micro-electromechanical systems (MEMSs), and batteries has made it possible to develop multirotor UAVs. Within the rotary-wing aircraft category, multirotor UAVs stand apart from helicopters primarily due to differences in the number and dimensions of their rotors. While helicopters use one or two main rotors to generate lift, with the primary one needing an additional tail rotor for stabilization [5], multirotor UAVs usually use pairs of rotors to generate lift force. The number of rotors and their arrangement depends upon the application of the UAV. Although multirotor UAVs lack the flight endurance of a conventional fixed-wing aircraft that is counterbalanced with the fast and precise flight positioning and maneuvering. A lot of research has been performed in the multirotor UAV field, and the possibilities of different configurations have been investigated [6,7,8]. The performance of the multirotor UAV derives from the propulsion configuration. By controlling the magnitude of the thrust force generated by the rotation of propellers, it is possible to achieve motion in 3D space. Multirotor UAVs are characterized as nonlinear and inherently unstable systems, necessitating the implementation of stabilization techniques. Integration of UAV technology into undergraduate education has focused on overcoming multirotor limitations [9], facilitating project-based learning [10], and offering open-source drone programming courses [11]. Due to their compact size and maneuverability, multirotors can be used as an excellent platform for the hands-on education of undergraduate mechatronics students. Their ability to be safely tested indoors makes them an ideal tool for practical learning, allowing students to experiment with control algorithms and understand the complexities of mechatronic systems in a controlled environment.

In addition to existing commercial UAVs that are often used in research and education [12,13,14], there are numerous studies in which custom solutions are presented, primarily focusing on quadrotors [15,16,17]. Some research highlights the use of multirotor UAV platforms, ranging from 2DOF drone testbeds to advanced 6DOF setups for control theory and stability validation [18,19,20,21]. From the perspective of control design, in addition to simulations and experimental tests, it is important to consider the hardware in the loop (HIL) experiments, such as the system based on Gazebo and Pixhawk flight controller (FC) presented in [22]. Control methods are embedded within the firmware that operates on the FC hardware. Open-source flight control software, including Dronecode (PX4 v1.13.3) [23], Ardupilot [24], Paparazzi UAV (v6.2.0) [25], and others, offer versatility in selecting the appropriate system for various applications. From the perspective of existing engineering education, integrating software solutions with current educational tools is essential. Additionally, emphasizing the importance of an intuitive experimental framework is crucial for effective software utilization in the educational context. MATLAB (MathWorks, Inc., Natick, MA, USA), a widely used tool in the field of control design, is demonstrated in the paper [26]. A variety of control and estimation methods are employed in research and product development [27,28,29,30,31], including conventional control methods such as Proportional–Integral–Derivative (PID) control or Linear Quadratic Regulator (LQR). This is of great significance from the educational point of view, as it can be incorporated into the classroom.

This paper serves as a comprehensive guide for educators on integrating multirotor UAVs into mechatronics education, specifically within undergraduate professional studies in Croatia, in alignment with the Croatian Qualification Framework (CQF)–HKO register. It provides a structured approach to utilizing UAVs as an educational tool while ensuring alignment with defined learning outcomes. The paper maps how UAV-based activities contribute to achieving specific learning objectives in mechatronics education and addresses three key aspects: mathematical modeling, hardware, and software.

One of these key aspects is mathematical modeling, where UAV dynamics are mathematically formulated and implemented in simulation environments to guide educators in achieving learning outcomes that enhance students’ theoretical understanding and practical application. Additionally, the paper explores the selection of suitable simulation software, such as MATLAB Simulink (R2021b), for effective modeling and analysis.

The second aspect, hardware, examines UAV component requirements, estimated costs, and potential upgrades based on budget constraints. To support this educational approach, an experimental UAV platform was developed using the PX4 ecosystem and MATLAB Simulink environment. This paper presents an educational prototype with airframe components that can be manufactured using additive manufacturing (AM) technologies, offering a cost-effective solution for integrating UAVs into the curriculum.

The final aspect, software, introduces a structured experimental framework that provides students with a hands-on learning experience. These experiments range from fundamental UAV control principles to advanced control strategies, conducted through laboratory work and interactive workshops. A series of control experiments were designed to enable students to apply control theory in real-world scenarios, refining their understanding through direct experimentation.

Three hardware-related learning outcomes were integrated into existing courses, while two software-related outcomes were introduced through student workshops aimed at refining educational materials. The proposed approach offers educators a scalable and flexible UAV-based learning framework adaptable to different institutional settings and budget constraints.

The paper is organized as follows: Section 2 presents the learning outcomes of the mechatronics undergraduate professional study program. Section 3 covers the mathematical modeling aspects of multirotor UAVs. The hardware aspects of multirotor UAVs are discussed in Section 4, while Section 5 focuses on the software aspects. The discussion and conclusions are presented in the final sections.

## 2. Mechatronics Undergraduate Professional Study Program in Croatia

UAVs are utilized across a broad spectrum of educational settings, as demonstrated in studies focusing on STEM education. For instance, STEM education for high school students can be conducted through workshops [32] or through other approaches, such as a mobile laboratory presented in [33], targets not only high school students but also elementary and middle school audiences. Additionally, various curricula have been developed, such as An Introductory Aeronautics Course [34], where high school students are introduced to the principles of quadrotor operation through lessons and laboratory exercises. In engineering education, the study of UAVs becomes more advanced, with the complexity of the material depending on whether it is taught at the undergraduate or graduate level. It is important to note that UAVs can be studied from multiple perspectives. Advanced topics in UAV studies often include mission planning for swarm of UAVs [35] or cooperative missions that involve other types of robots [36]. As with other systems, the integration of hardware and artificial intelligence unlocks numerous possibilities [37,38,39].

This paper examines the undergraduate professional study of mechatronics in Croatia, aligned with the HKO register and qualification standards. The Mechatronics undergraduate professional study program has been implemented since 2021 [40]. The program leads to a professional bachelor’s degree (baccalaureus/baccalaurea) in mechatronics engineering. The program has a total of 180 ECTS points, of which 114 ECTS points are allocated to mandatory learning outcomes, and 66 ECTS points to optional learning outcomes. Table 1 presents the learning outcomes of the mechatronics study program. Learning outcomes IUP1 to IUP10 belong to general learning outcomes (soft skills), while the remaining learning outcomes (IUP11 to IUP27) belong to professional learning outcomes.

From the perspective of general learning outcomes, all of them correspond, at least slightly, partially, or strongly, with multirotor UAVs. The paper will specifically explore IUP1 and IUP6, illustrating how the integration of a multirotor UAV as an educational tool enhances comprehensive skill acquisition and provides a holistic learning experience for students. Regarding professional learning outcomes, outcomes not related to multirotor UAVs (IUP19, IUP20, IUP22) will not be considered further. All other professional outcomes are either partially or strongly associated with multirotor UAVs. It can be freely written that multirotor UAVs serve as a versatile platform for teaching various aspects of mechatronic engineering due to their multidisciplinary nature. By working with this platform, students can apply theoretical concepts learned in the classroom to real-world scenarios and gain practical skills that are highly valued in the industry.

**Figure 1:**
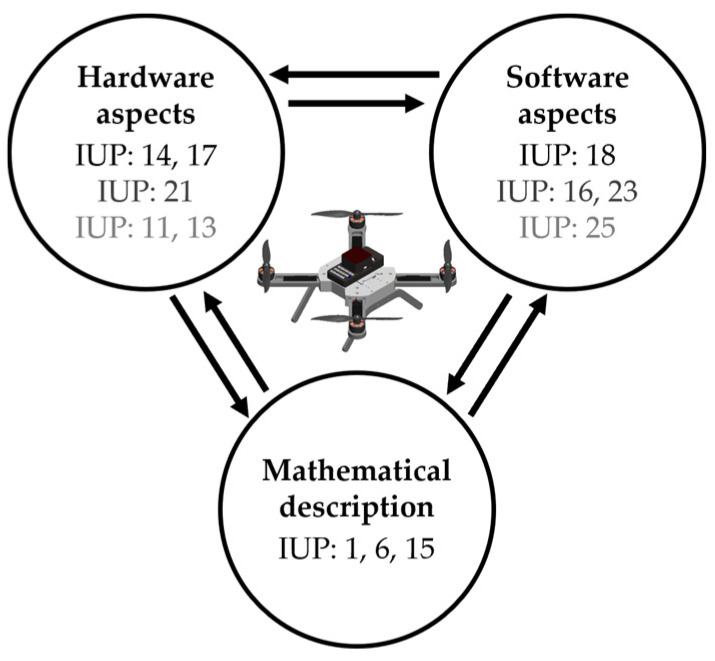
Grouped learning outcomes of the professional undergraduate study of mechatronics.

Considering the aforementioned general learning outcomes as well as the professional ones that align closely with the multirotor UAVs, a categorization into three primary aspects is proposed, as illustrated in Figure 1. Each aspect is associated with specific learning outcomes. Furthermore, separate sections will provide a detailed description of the learning outcomes (IUP: 1, 6, 14, 15, 16, 17, 18, 21, 23) that are partially or strongly related to multirotor UAVs, and teaching modules will be proposed in the form of auditory, laboratory, or construction exercises. Additionally, other outcomes depicted in the scheme (IUP: 11, 13, 25) will be briefly mentioned and linked to multirotor UAVs.

## 3. Multirotor UAV Mathematical Description

In this section, the multirotor UAV system is briefly described and linked to the learning outcomes IUP1, IPU6, and IUP15. Starting with IUP6, the multirotor UAV serves as an ideal platform for fostering student learning, promoting problem-solving, and enhancing critical thinking skills. These benefits arise from the inherent properties of the multirotor UAV, regardless of their configuration, with three key characteristics being particularly significant:

- Inherently unstable system
- Highly nonlinear system
- Multivariable system

The inherent instability of multirotor UAVs poses a significant challenge, especially regarding control. In the event of a control loop malfunction, the multirotor UAV loses its ability to autonomously maintain a balanced state (hovering). Instead, it begins to move uncontrollably, and without restoration of the control loops, it will eventually descend and crash. Therefore, selecting the appropriate components and software solutions for the control system is crucial. To achieve this objective, it is essential to acquire a comprehensive understanding of UAV operational principles, including parameter identification and system modeling. The mathematical model of a multirotor UAV will be described using common tools and methods accessible to students in the second and third years of the course in professional mechatronics engineering. The model can be divided into three parts: the kinematic model, the dynamic model, and the allocation model of the multirotor propulsion configuration. Applying mathematical and physical laws (for example IUP1) is essential for effectively addressing engineering challenges, as it involves selecting the appropriate tools and methods needed for successful problem-solving. The following three subsections will demonstrate the application of mathematical and physical laws in the mathematical description of multirotor UAVs, which can be used as teaching materials in mechatronics courses.

**Figure 2:**
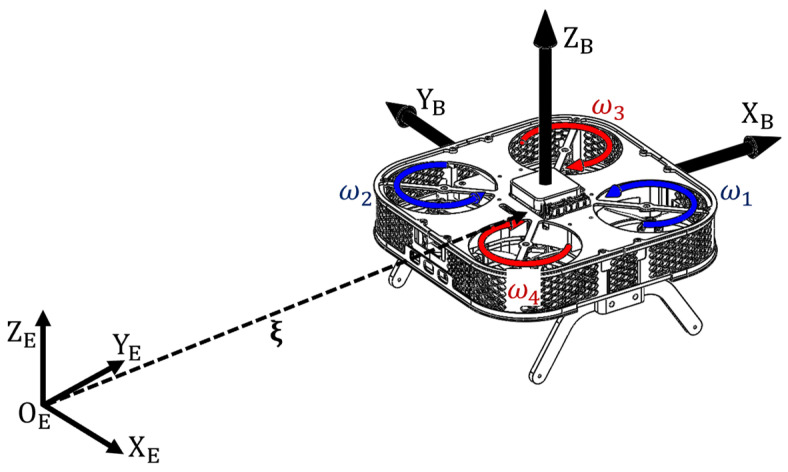
Quadrotor UAV reference coordinate systems.

For modeling purposes, the multirotor UAV can be represented schematically as shown in Figure 2. The figure shows the two main coordinate systems (frames) required to define the mathematical model of a multirotor UAV. The first frame, known as the inertial frame or the earth reference frame ($F^{E}$ {$O_{E},X_{E},Y_{E},Z_{E}$}), is fixed in time and space. The state of the aircraft in real time is determined by its position, $\xi ={\left[\begin{array}{ccc} x & y & z \end{array}\right]}^{T}$, and orientation, $\eta ={\left[\begin{array}{ccc} \phi & \theta & \psi \end{array}\right]}^{T}$, in 3D space, with respect to the $F^{E}$, which depends on the mission profile. The second frame is anchored at the UAV’s center of gravity and moves in sync with the aircraft, with a dynamic model affixed to it. This frame is commonly referred to as the body frame ($F^{B}$ {$O_{B},X_{B},Y_{B},Z_{B}$}). Since the dynamics of the aircraft are defined relative to the body frame, the aircraft’s acceleration vector is given by $\dot{\nu }={\left[\begin{array}{cc} \begin{array}{ccc} \dot{u} & \dot{v} & \dot{w} \end{array} & \begin{array}{ccc} \dot{p} & \dot{q} & \dot{r} \end{array} \end{array}\right]}^{T}$, from which translational velocity $v={\left[\begin{array}{ccc} u & v & w \end{array}\right]}^{T}$ and rotational velocity $\omega ={\left[\begin{array}{ccc} p & q & r \end{array}\right]}^{T}$ relative to the body frame are determined.

### 3.1. UAV Kinematics Model

According to Euler’s orientation theorem, any complex rotation can be decomposed into elementary rotations ($R^{3\times 3}$) about known axes. Euler’s theorem can be applied under the assumption that the multirotor UAV is represented as a rigid body, where the only moving parts are the rotors of the aircraft. The rotation of a rigid body is described by three consecutive rotations, defined by the following rotation matrices:

(1) $$R\left(\psi ,\;Z\right)=\left[\begin{array}{ccc} c_{\psi } & s_{\psi } & 0\\ {-s}_{\psi } & c_{\psi } & 0\\ 0 & 0 & 1 \end{array}\right],\;R\left(\theta ,Y_{1}\right)=\left[\begin{array}{ccc} c_{\theta } & 0 & {-s}_{\theta }\\ 0 & 1 & 0\\ s_{\theta } & 0 & c_{\theta } \end{array}\right],\;R\left(\phi ,\;X_{B}\right)=\left[\begin{array}{ccc} 1 & 0 & 0\\ 0 & c_{\phi } & s_{\phi }\\ 0 & {-s}_{\phi } & c_{\phi } \end{array}\right].$$

here, $c_{\alpha }=\cos\left(\alpha \right)$ and $s_{\beta }=sin\;(\beta )$. These rotation matrices are applied using the auxiliary coordinate systems positioned between the inertial and body frames, with axes labeled as $X_{1}$, $Y_{1}$, and $Z_{1}$. A more detailed description and a schematic representation of these coordinate transformations are provided in the paper [41]. The rotation matrices are orthogonal ($R^{-1}=R^{T}$).

From a modeling perspective, the kinematic model can be divided into translational and rotational kinematics. The translational part maps values from the body frame to the inertial (fixed) frame using the expression $\dot{\xi }=R_{B}v$. Based on the order of rotations (yaw-pitch-roll), the overall rotation matrix $R_{B}=R^{T}\left(\psi ,Z\right)R^{T}\left(\theta ,Y_{1}\right)R^{T}\left(\phi ,X_{B}\right)$ is:

(2) $$R_{B}=\left[\begin{array}{ccc} c_{\psi }c_{\theta } & c_{\psi }s_{\theta }s_{\phi }{-s}_{\psi }c_{\phi } & c_{\psi }s_{\theta }c_{\phi }+s_{\psi }s_{\phi }\\ s_{\psi }c_{\theta } & s_{\psi }s_{\theta }s_{\phi }+c_{\psi }c_{\phi } & s_{\psi }s_{\theta }c_{\phi }{-c}_{\psi }s_{\phi }\\ -s_{\theta } & c_{\theta }s_{\phi } & c_{\theta }c_{\phi } \end{array}\right].$$

Rotation matrices are also used to describe the following rotational kinematics of the multirotor UAV, which maps rotational velocities from the body frame to the earth frame using the relationship $\dot{\eta }=T_{B}\omega$. This mapping involves a complex transformation matrix, whose derivation can also be applied in engineering education. A more detailed explanation can be found in the paper [41]. The transformation matrix is given:

(3) $$T_{B}=\left[\begin{array}{ccc} 1 & s_{\phi }t_{\theta } & c_{\phi }t_{\theta }\\ 0 & c_{\phi } & -s_{\phi }\\ 0 & s_{\phi }/c_{\theta } & c_{\phi }/c_{\theta } \end{array}\right],$$

where $t_{i}=\tan\left(i\right)$. Based on the rotation and transformation matrices, the main kinematic equation for UAVs, which maps the velocities from the body frame to the earth frame, is given by:

(4) $$\dot{\varepsilon }=\left[\begin{array}{cc} R_{B} & 0_{3\times 3}\\ 0_{3\times 3} & T_{B} \end{array}\right]\nu .$$

To fully establish the kinematics model, it is necessary to apply integration methods. This is necessary because the input to the kinematics model comprises the aircraft’s acceleration relative to the body frame, which results from the aircraft’s dynamics. Additionally, the integration of velocities obtained in the earth frame is required to determine the aircraft’s position and orientation in 3D space. Existing integration methods available in software packages can be utilized to conduct simulations, offering further exploration opportunities in engineering education.

Following this section of the model, the utilization of fundamental rotation matrices is of critical importance in mechatronic engineering, especially in describing the kinematics of multirotor UAVs and defining the aircraft’s configuration. Beyond UAV kinematics, these matrices are also used in modeling robotic arms with rotary joints (DOFs) and various other applications.

### 3.2. Multirotor UAV Dynamics Model

Similarly to its kinematics, the dynamics of a multirotor UAV are also described by mathematical and physical laws (IUP1). After defining and establishing the kinematic model under the rigid body assumption, the dynamic model is developed by introducing forces and moments in subsequent stages of the modeling. This model describes the multirotor UAV as a rigid body governed by six second-order differential equations. The equations of motion are derived by applying the basic kinematic equation that connects the derivative of a vector between two coordinate systems. In its general form, this relationship is expressed as:

(5) $$\frac{DQ}{Dt}=\frac{dQ}{dt}+\omega \times Q,$$

where ω is the angular velocity vector of the rotating coordinate frame relative to the fixed coordinate frame. Euler’s laws of motion extend Newton’s laws to rigid body motion. Accordingly, the equations of motion were therefore derived using the Newton-Euler method, under the assumption that the origin of the body frame coincides with the center of gravity and that its axes align with the principal axes of inertia of the UAV. To ensure compliance with this assumption during the design and construction of a multirotor UAV, the assembly should be symmetrical, with the mass of its components and parts concentrated at the center of the UAV.

To enhance students’ understanding of the model, similar to the kinematic approach, the equations of motion, and consequently the dynamic model, are divided into translational and rotational parts. The translational (linear) motion of a rigid body, based on Euler’s first law and derived using the basic kinematic equation, is given by the expression:

(6) $$m\frac{dv}{dt}+\omega \times \left(mv\right)=F,$$

where m is the total mass of the UAV (including payload), which is time-invariant, except in cases involving payload transfer or dispersal, where the mass becomes time-variable. On the right-hand side of the equation, F represents the vector of all forces acting on the UAV’s motion in 3D space, given by $F={\left[\begin{array}{ccc} F_{X} & F_{Y} & F_{Z} \end{array}\right]}^{T}$. These forces encompass the total external forces influencing the UAV’s movement.

The rotational (angular) motion of a rigid body, based on Euler’s second law, is given:

(7) $$I\frac{d\omega }{dt}+\omega \times \left(I\omega \right)=T,$$

where I is the body’s inertia tensor, which depends on the mass distribution and arrangement of the UAV’s components with respect to the body frame $F^{B}$. Like the mass, the inertia is assumed to be time-invariant. To simplify the model, the UAV’s body is assumed to be symmetrical with respect to its principal axes of inertia, resulting in the inertia tensor being represented by a diagonal matrix $I=diag\left\{\begin{array}{ccc} I_{xx}, & I_{yy}, & I_{zz} \end{array}\right\}$. On right-hand side of Equation (7), T is the vector of moments acting on the UAV’s rotational motion, expressed as $T={\left[\begin{array}{ccc} T_{\phi } & T_{\theta } & T_{\psi } \end{array}\right]}^{T}$. These moments correspond to the torques around the UAV’s principal axes.

To facilitate the simulation implementation, the model can undergo additional reformulation through the application of mathematical laws, thereby enriching the inclusion of practical examples within IUP6. Vector products in the equations of motion can be represented in matrix form using a skew-symmetric matrix (a×b=S(a)b), which simplifies the integration of the model into the software package. The matrix representation of the equations of motion governing all six DOF of the multirotor UAV can be expressed as:

(8) $$M_{B}\dot{\nu }+C_{B}\left(\nu \right)\nu =\Lambda .$$

Matrix $M_{B}$ contains parameters that provide resistance to the body’s motion, with translational dynamics affected by the mass and rotational dynamics by the moments of inertia. Under the aforementioned assumptions, this matrix can be simplified to a diagonal form $M_{B}=diag\left\{\begin{array}{cccccc} m, & m, & m, & I_{xx}, & I_{yy}, & I_{zz} \end{array}\right\}$. Given that the UAV can freely rotate in 3D space, the Coriolis and centripetal matrix $C_{B}\left(\nu \right)$ describes the inertial forces and moments. For simulation purposes, it is assumed that the body frame is aligned with the center of gravity and the UAV’s axes. Additional information on the derivation of the $C_{B}\left(\nu \right)$ matrix and parameterization methods are provided in the literature [42].

The right side of Equation (8) represents the combined vector of forces and moments $\Lambda ={\left[\begin{array}{cc} \begin{array}{ccc} F_{X} & F_{Y} & F_{Z} \end{array} & \begin{array}{ccc} T_{\phi } & T_{\theta } & T_{\psi } \end{array} \end{array}\right]}^{T}$, which influences the aircraft’s dynamics across all six DOFs. These forces and moments originate from both environmental factors and the aircraft’s propulsion system. Regarding the environment, gravitational force holds primary significance, while additional effects such as air resistance caused by aircraft movement and influences like wind gusts can be modeled for simulation purposes. Moreover, propulsion forces and moments from the propulsion system directly influence aircraft dynamics, constituting the control vector. While effects like the gyroscopic effect on rotational dynamics can also be modeled, they may be disregarded in simplified models and included as unmodeled dynamics alongside external disturbances in the overall disturbance vector. Furthermore, a simplified dynamics model will be presented, which is planned for implementation in the program package and simulations related to IUP15.

In the simplified model, the components of gravitational force, propulsion forces, and moments are included, while the other listed effects will be incorporated into the disturbance vector as non-modeled dynamics. It is well-known that gravitational force acts along the z-axis in the earth reference frame ($F^{E}$). Considering the East, North, Up (ENU) convention used for the model, as shown in Figure 2, the gravitational force with respect to the earth frame is equal to $g_{E}={\left[\begin{array}{ccc} 0 & 0 & -mg \end{array}\right]}^{T}$, where g denotes the gravitational constant. Since the aircraft’s dynamics occur in relation to the body frame, rotation matrices are employed to map the gravitational force, as depicted in the expression $g_{B}={R_{B}}^{T}g_{E}$. Gravitational force exclusively impacts translational dynamics, while propulsion system forces ${f=\left[\begin{array}{ccc} f_{X} & f_{Y} & f_{Z} \end{array}\right]}^{T}$ influence translational dynamics from a control perspective. Unmodeled dynamics and external disturbances are contained in the vector $d_{f}={\left[\begin{array}{ccc} d_{X} & d_{Y} & d_{Z} \end{array}\right]}^{T}$. Furthermore, rotational dynamics are affected by the moments of the propulsion system $\tau ={\left[\begin{array}{ccc} \tau_{\phi } & \tau_{\theta } & \tau_{\psi } \end{array}\right]}^{T}$, while the other effects are included in the vector $d_{\tau }={\left[\begin{array}{ccc} d_{\phi } & d_{\theta } & d_{\psi } \end{array}\right]}^{T}$. The vector of forces f and moments τ of the propulsion configuration form the control vector $u_{B}={\left[\begin{array}{cc} \begin{array}{ccc} f_{X} & f_{Y} & f_{Z} \end{array} & \begin{array}{ccc} \tau_{\phi } & \tau_{\theta } & \tau_{\psi } \end{array} \end{array}\right]}^{T}$. In view of the above, the acceleration vector with respect to the body frame can be described by the following expression

(9) $$\dot{\nu }={M_{B}}^{-1}(-C_{B}\left(\nu \right)\nu +g_{B}+u_{B}-d),$$

### 3.3. Multirotor UAV Configuration Allocation Model

The versatility of multirotor UAVs makes them extremely interesting in engineering education, as they enhance understanding and knowledge. Additionally, students learn to apply mathematical modeling and simulation techniques to analyze and optimize UAV performance, which aligns with IUP1 and IUP6. Mathematically, a multirotor is a multivariable system composed of *N* rotors responsible for providing propulsion forces and moments. To perform the mission, it is necessary to primarily select the parameters of the propulsion configuration, which are described from the dynamic perspective as forces f and torques τ with respect to the body frame. Given that multirotor aircraft are characterized by high energy consumption, selecting the propulsion parameters is crucial for increasing system efficiency.

**Figure 3:**
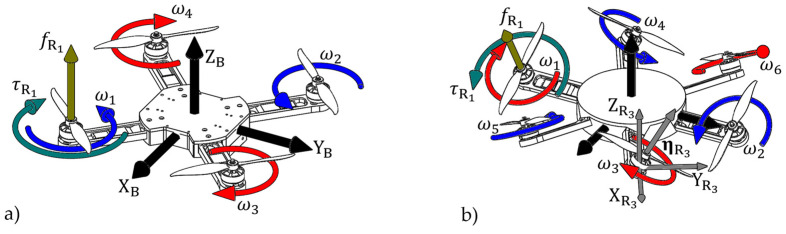
Multirotor UAV propulsion configurations: (**a**) Conventional quadrotor configuration; (**b**) Fully actuated hexarotor configuration.

To illustrate a more general model of the multirotor propulsion configuration, Figure 3 presents both the conventional and fully actuated configurations of the multirotor UAV, which are increasingly used. The propulsion configuration is determined by the geometric arrangement of the rotors and their characteristics. Rotor characteristics depend on the components, and conventional rotors that make up electric propulsion units (EPUs) are further considered. In modeling the propulsion configuration, it is assumed that the rotors of the electric motor, on which the propeller is mounted, produce aerodynamic effects through their motion. The thrust force $f_{R_{i}}$ and the drag torque $\tau_{R_{i}}$ of the *i*-th rotor are defined as follows. Thrust forces form the vector $f_{R}={\left[\begin{array}{cccc} f_{R_{1}} & f_{R_{2}} & \ldots & f_{R_{N}} \end{array}\right]}^{T}$ and drag torques are represented by the vector $\tau_{R}={\left[\begin{array}{cccc} \tau_{R_{1}} & \tau_{R_{2}} & \ldots & \tau_{R_{N}} \end{array}\right]}^{T}$.

Given that the dynamics of the UAV are defined with respect to the body frame, it is necessary to map the forces and torques of the propulsion configuration to the control vector $u_{B}$. For this purpose, through further modeling, the geometric arrangement of the configuration is defined using the vector of the position $\xi_{R_{i}}$ and orientation $\eta_{R_{i}}$ of the *i*-th rotor with respect to the body frame. The application of rotation matrices is repeated in this part of the model, as described in more detail in the paper [41]. A matrix which contains the orientations of individual rotors $H=\left[\begin{array}{ccc} \eta_{R_{1}} & \cdots & \eta_{R_{N}} \end{array}\right]$ is used to map thrust forces $f_{R}$ to the propulsion system force vector f and to map drag torques $\tau_{R}$ to the propulsion system moment vector τ. The vector of moments of the propulsion system also include components related to thrust forces, as the arrangement of the rotors also creates moments. Mapping can be defined by applying a vector product, but a more elegant approach involves using a matrix representation, where matrix $\Xi =\left[\begin{array}{ccc} S\left(\xi_{R_{1}}\right)\eta_{R_{1}} & \cdots & S\left(\xi_{R_{N}}\right)\eta_{R_{N}} \end{array}\right]$ maps the thrust forces to the vector of moments of the propulsion system. Consequently, the matrix expression that describes the mapping of thrust forces and drag torques to the control vector of the multirotor UAV is:

(10) $$u_{B}=\left[\begin{array}{c} H\\ \Xi \end{array}\right]f_{R}+\left[\begin{array}{c} 0_{3\times N}\\ H \end{array}\right]\tau_{R},$$

Conventional multirotor configurations generally consist of an even number of identical rotors symmetrically arranged in one or more parallel planes. Each pair of rotors consists of clockwise (CW) and counterclockwise (CCW) rotors to cancel the reactive moment about the vertical axis of the aircraft. The most commonly used configuration has four rotors (Figure 3a), the so-called quadrotor (quadcopter). In the planar arrangement of the rotors, the orientation vectors of all rotors are unit vectors. The sign of the rotor drag torques depends on the direction of rotation: CW rotors have a positive sign, while CCW rotors have a negative sign. The mapping of thrust force vectors and drag torques for the quadrotor configuration with an X arrangement is given by the following expression:

(11) $$u_{B}=\left[\begin{array}{c} u_{1}\\ u_{2}\\ u_{3}\\ u_{4} \end{array}\right]=\left[\begin{array}{cccc} 1 & 1 & 1 & 1\\ -\frac{\sqrt{2}}{2}l & \frac{\sqrt{2}}{2}l & \frac{\sqrt{2}}{2}l & -\frac{\sqrt{2}}{2}l\\ -\frac{\sqrt{2}}{2}l & \frac{\sqrt{2}}{2}l & -\frac{\sqrt{2}}{2}l & \frac{\sqrt{2}}{2}l\\ 0 & 0 & 0 & 0 \end{array}\right]\left[\begin{array}{c} f_{R_{1}}\\ f_{R_{2}}\\ f_{R_{3}}\\ f_{R_{4}} \end{array}\right]+\left[\begin{array}{cccc} 0 & 0 & 0 & 0\\ 0 & 0 & 0 & 0\\ 0 & 0 & 0 & 0\\ 1 & 1 & 1 & 1 \end{array}\right]\left[\begin{array}{c} \tau_{R_{1}}\\ \tau_{R_{2}}\\ \tau_{R_{3}}\\ \tau_{R_{4}} \end{array}\right],$$

where l is the rotor arm length, the distance from the origin of the body frame to the rotor’s z-axis. The first control variable $u_{1}$ represents the control force along the z-axis of the body frame $f_{Z}$, while the second control variable $u_{2}$ corresponds to the moment $\tau_{\phi }$, the third $u_{3}$ to the moment $\tau_{\theta }$, and the fourth $u_{4}$ to the moment $\tau_{\psi }$. Given that the orientation vectors of each rotor in planar configurations are unit vectors, the size of the control vector is four. In these cases, multirotor UAVs represent underactuated systems with coupled dynamics since the control vector size is less than the number of UAV DOFs. This applies to all planar configurations, regardless of the number of rotors, whether quadrotor (four rotors), hexarotor (six rotors), or octorotor (eight rotors) UAVs.

From the perspective of EPU characteristics, a model will be employed for simulations where the thrust force is described as a quadratic function dependent on the rotor’s angular speed. The thrust force of the *i*-th rotor is given by the expression:

(12) $$f_{R_{i}}=k_{f_{i}}{\omega_{i}}^{2},$$

where $k_{f_{i}}$ is the thrust force factor dependent on the propeller geometry, air density, and interference, while $\omega_{i}$ is the angular velocity of the *i*-th rotor, representing the model input. The following aerodynamic effect is the drag torque, which for the *i*-th rotor is given by the expression:

(13) $$\tau_{R_{i}}=k_{\tau_{i}}{\omega_{i}}^{2},$$

where $k_{\tau_{i}}$ is the drag torque factor of the *i*-th rotor, with the sign of the factor depending on the rotation direction of the rotor. Using Equations (11)–(13), a simplified and standard representation of the mapping of thrust forces and drag torques for a quadrotor configuration can be shown, where model input is represented by the angular velocities of the rotors:

(14) $$u_{B}=\left[\begin{array}{c} u_{1}\\ u_{2}\\ u_{3}\\ u_{4} \end{array}\right]=\left[\begin{array}{cccc} k_{f} & k_{f} & k_{f} & k_{f}\\ -\frac{\sqrt{2}}{2}k_{f}l & \frac{\sqrt{2}}{2}k_{f}l & \frac{\sqrt{2}}{2}k_{f}l & -\frac{\sqrt{2}}{2}k_{f}l\\ -\frac{\sqrt{2}}{2}k_{f}l & \frac{\sqrt{2}}{2}k_{f}l & -\frac{\sqrt{2}}{2}k_{f}l & \frac{\sqrt{2}}{2}k_{f}l\\ -k_{\tau } & -k_{\tau } & k_{\tau } & k_{\tau } \end{array}\right]\left[\begin{array}{c} {\omega_{1}}^{2}\\ {\omega_{2}}^{2}\\ {\omega_{3}}^{2}\\ {\omega_{4}}^{2} \end{array}\right].$$

Apart from conventional configurations widely employed across industries, various unconventional setups, mainly with non-planar rotor geometric arrangements, have been explored and implemented. The versatility of propulsion configurations offers opportunities for enhancement, whether it is boosting efficiency, as demonstrated in the paper [43], or increasing the degree of actuation [44]. Equation (10) reveals that the degree of actuation depends on the rotor orientation matrix. One method to achieve full actuation involves tilting the rotors along the axis of their arms, resulting in so-called passively tilted multirotor configurations (Figure 3b). As multirotor UAVs are increasingly employed as aerial robots, they are assigned missions that demand precise and intricate maneuvers. Consequently, fully actuated configurations become notably intriguing as they contribute to a deeper understanding of the system and its capabilities.

Returning to the dynamic model, the parameters of the rigid body depend on the parameters and components of the propulsion configuration. In addition to batteries, the components and the airframe of the propulsion configuration significantly influence the total mass of the aircraft and the inertia tensor. As for determining the parameters, the mass is easy to find, but the moments of inertia can be determined either theoretically through computation or practically using software. This paper will focus on the practical approach, particularly in relation to 3D modeling, which is also associated with the learning outcomes.

### 3.4. Model Implementation and Simulation Framework

**Figure 4:**
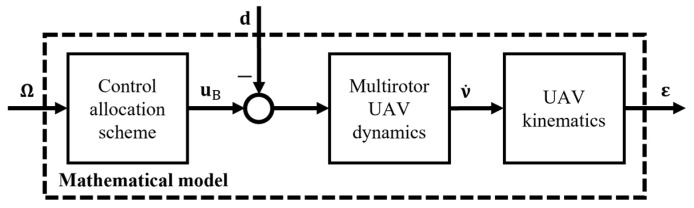
Schematic representation of the multirotor UAV mathematical description.

Once all the considered elements of the multirotor UAV mathematical model have been established, the next step directly aligns with the learning outcome IUP15 (Analyze the behavior of mechatronic systems by modeling and simulating). An implementation and simulation framework for multirotor UAV models is crucial in engineering education. It provides students with a comprehensive understanding of both the theoretical and practical aspects of UAV design and operation. Additionally, the general learning outcome IUP9 (Use techniques, skills, and modern tools essential for engineering practice) can be highlighted here. Given its widespread application across various courses, the MATLAB Simulink (R2021b) software package is particularly suitable for use with a multirotor platform. It facilitates the execution of simulations as well as experiments, making it ideal for both classroom and hands-on education. Figure 4 shows a schematic mathematical description of the multirotor UAV, which is implemented through a software package and, alongside the control module, forms a closed loop.

**Figure 5:**
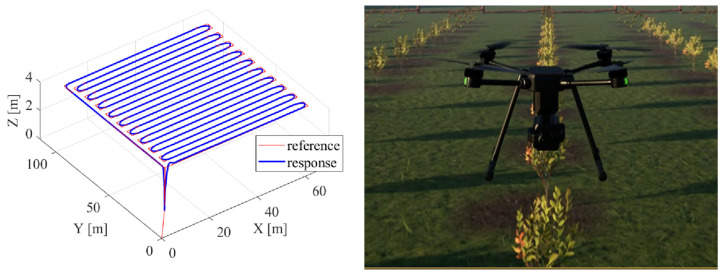
Simulating the behavior of a multirotor UAV for a typical task.

It is important to emphasize that, due to the characteristics of such platforms, safety is a critical consideration in practical engineering education. This framework allows students to engage with kinematic and dynamic models, gaining insight into the real-world behaviors and challenges of aerial systems. The ability to simulate various scenarios, such as precision agriculture missions (Figure 5), enhances problem-solving skills and prepares students to meet industry demands. The simulation framework supports solving problems in various areas, such as system design for efficiency, control design, path planning, and more, thus equipping future engineers with the necessary tools and knowledge to innovate and succeed in the field of autonomous aerial systems.

## 4. Multirotor UAV Hardware Aspects

In general, multirotor UAVs can be divided into several essential subsystems that function as modules, i.e., the control (avionics), propulsion, and energy modules, along with landing gear, equipment, and payload. The components of the control module are crucial when incorporating the multirotor UAV into engineering education. In this regard, the PX4 ecosystem emerges as an excellent platform for engineering education due to its seamless integration with the MATLAB programming environment, as mentioned earlier. Of particular significance is the FC, and for this research, the Cube Black, Cube Orange, and Cube Purple, along with their corresponding carrier boards, were utilized as central components. The main disadvantage of using the Cube FC series is its cost, which exceeds the combined cost of all other parts and components, excluding the position control modules. Alternatively, other open-source FCs can be used, provided their ecosystems can be integrated within education. In cases where MATLAB cannot be used, for example, due to licensing restrictions, the development process can shift towards using traditional microcontroller programming tools, like PlatformIO or Python-based frameworks such as MicroPython. These options are cost-effective, widely supported, and ideal for embedded systems development. Simulink alternatives like Scilab also provide free, open-source simulation environments. The decision to choose these alternatives over MATLAB is often driven by budget constraints, project-specific requirements, or the need for open-source compatibility. Each of these tools has distinct advantages, such as MATLAB’s robust simulation environment and integration capabilities, while open-source alternatives emphasize accessibility and adaptability in resource-constrained settings.

**Figure 6:**
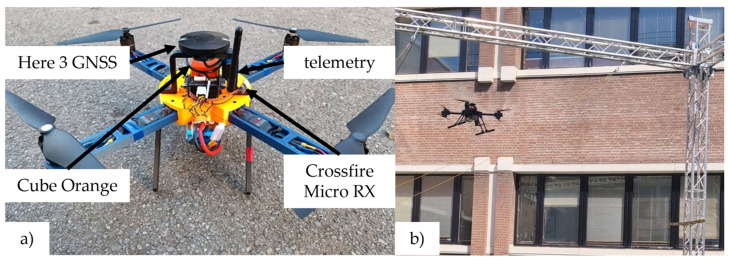
Multirotor UAV hardware: (**a**) Control system components; (**b**) Multirotor testing.

The Cube series of FC enables the connection of various peripheral modules and sensors such as GNSS and telemetry. Furthermore, two types of remote-control (RC) subsystems were analyzed and used in this research for prototype platform testing and experimental implementation. The first type operates on a standard 2.4 GHz frequency, using the FrSky X8R receiver to access all 16 channels via the SBUS RC protocol, utilizing Taranis X9s and X7s remote control transmitters. The second type consists of TBS Crossfire receivers that operate at a lower frequency (868 MHz), also using the SBUS RC protocol. In this setup, a TX16S transmitter is used in combination with a Crossfire RC link. The FC carrier board supplies peripheral components and sensors and is powered via a power brick with a sensor connected between the batteries and the propulsion components, providing a stable 5 V voltage. It is used for consumption up to 60 A at 2S, 3S, and 4S voltage setups. Figure 6a shows a prototype multirotor aircraft equipped with an avionics module based on the Cube Orange FC and its associated components. For educational purposes, ensuring a safe environment for outdoor aircraft testing is important. This can be achieved by using a secure enclosure, such as the cage depicted in Figure 6b, for position control tests of the prototype aircraft.

In recent years, enrollment in the second and third years of the undergraduate professional study program in Mechatronics in Karlovac has ranged between 20 and 25 students. This number will be considered when procuring components. For laboratory exercises, students will be divided into groups of up to five, as the plan is to produce five experimental platforms. Among these five UAV platforms, in terms of position control, two will be equipped with GNSS modules for outdoor flight, while three will feature companion computers (initially, Raspberry Pi 5 microcomputers are planned for procurement). This paper considers indoor position control due to the fact that the UAV Laboratory in Karlovac is equipped with a motion capture system. Table 2 outlines the mandatory and optional components depending on the type of control. Checkmarks in parentheses indicate components that are optional for specific experiments.

Two propulsion sets, the AirGEAR 200 and AirGEAR 355, are under consideration. Each set comprises four propulsion units, with prices ranging from approximately €80 to €100. These will be paired with batteries of 1800–3000 mAh capacity, priced between €20 and €30 each. The cost of airframe components also varies depending on the UAV’s size, which is determined by the selected propulsion components, and ranges from €20 to €30. The most expensive component of the UAV, as previously mentioned, is the FC, along with its associated elements, including the carrier board, telemetry, and power module with a sensor. Collectively, these components cost approximately €300. Additionally, remote control (RC) systems are planned for all five UAVs, with the onboard receiver (Radiomaster R88 V2 LBT Receiver) and remote control transmitter (RadioMaster Pocket) priced at approximately €70.

**Figure 7:**
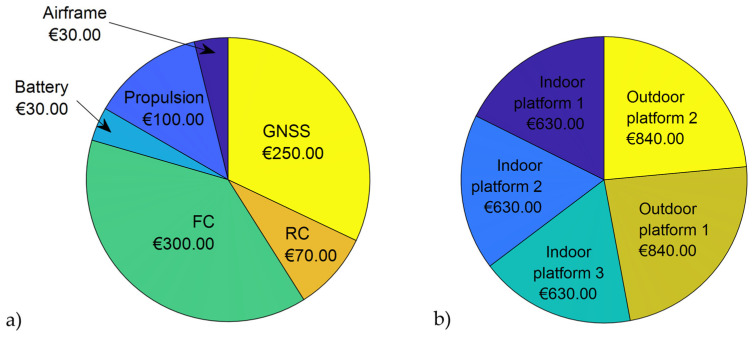
Multirotor UAV hardware cost: (**a**) Cost share of modules for outdoor platform; (**b**) Cost share for five multirotor platforms.

For laboratory exercises in the Fundamentals of Automatic Control and Robotics courses, the table outlines the mandatory and optional components and modules required for conducting experiments. Basic experiments include the synthesis of control algorithms and the tuning of controller parameters (attitude control experiments in Fundamentals of Automatic Control), as well as demonstrations of system operation principles and piloting in stabilized mode using inertial sensors (remote control experiments in Robotics). The cost of components for these basic experiments ranges from approximately €490 to €530, with the FC and its associated components comprising the largest share of the cost. To expand the functionality of the multirotor UAV platform, position control experiments will also be implemented. For outdoor platform configurations, an additional GNSS module, priced at approximately €250, increases the total cost of the platform to approximately €840–€880. Assuming the availability of an OptiTrack system, indoor position control experiments will utilize a companion computer, costing approximately €100. A graphical representation of the cost breakdown for individual modules and components for outdoor position control is provided in Figure 7a. Additionally, the share of the total cost for assembling three indoor platforms and two outdoor platforms is illustrated in Figure 7b. Considering the approximate costs, we can state that the procurement of five platforms (two for outdoor use and three for indoor use) amounts to approximately €3500.

### 4.1. Propulsion Configuration Hardware

The propulsion configuration (module) in many ways defines multirotor UAV. Conventional configurations consist of an even number of symmetrically placed EPUs. The description of propulsion configurations, especially EPUs, is directly aligned with IUP14 (To know the principles of operation of electronic and electromechanical converters). Since the propulsion components are responsible for generating the necessary aerodynamic forces and torques, the characteristics of EPUs directly link the mathematical description with the aircraft’s hardware. Therefore, this section will describe how the multirotor propulsion hardware can be utilized as a hands-on learning tool. For educational purposes, multirotor UAVs with a diameter (diagonal) of up to 0.5 m have been considered, making them suitable for indoor experiments. To propel an electric multirotor UAV, EPUs consist of a Brushless DC (BLDC) electric motor, an Electronic Speed Controller (ESC), and a fixed-pitch propeller mounted on a motor rotor. A wide range of concepts can be incorporated into the educational process using multirotor EPUs as a case study, with hands-on exercises provided for demonstration.

The BLDC motor is a type of permanent magnet electric motor where electromagnets (armature) are located on the motor’s stator, while permanent magnets are located on the rotor, which is positioned outside the stator. This configuration is known as an outrunner motor. The motor is driven by a rectangular-shaped input voltage (six-step commutation) provided by an ESC, which converts the supplied DC voltage into appropriate motor phase voltages. The desired RPM is established by the input PWM signal originating from the FC. Considering the operating principle of the multirotor propulsion configuration, which includes an even number of CW and CCW EPUs, it is essential to correctly connect the phases of the BLDC motor and the ESC. This step is crucial in hardware testing, as the motor’s rotation direction determines the appropriate propeller geometry (CW or CCW).

**Figure 8:**
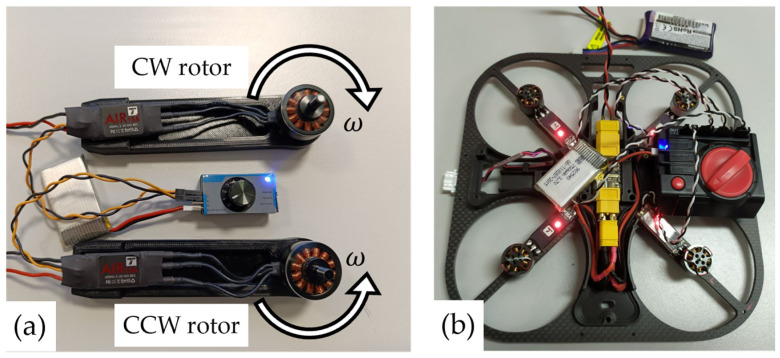
Propulsion hardware testing: (**a**) EPU rotation adjustment; (**b**) Propulsion calibration.

The procedure for implementing hands-on teaching is described to ensure that students can safely perform the experiments. In an educational setting, this experiment involves connecting the BLDC motor phases to the ESC phases, as depicted in Figure 8a, while ensuring that the ESCs are unpowered and the propellers are not mounted on the BLDC rotor. After connecting the phases (either with connectors or by soldering), the ESC is then connected to the control signal to set the rotor’s angular velocity. This signal can be provided through FC, RC, an experimental setup, or, in simpler cases, via a servo tester. Initially, the control signal must be set to its minimum value. Next, the ESC is connected to the power source (battery). Upon connection, the EPU emits characteristic sounds, signaling its readiness for operation or indicating any errors. If the motor is ready for operation, the direction of rotation can be determined by gradually increasing the control signal.

The second part of this experiment involves calibrating the ESC, which is crucial for multirotor flight. This calibration is performed according to the ESC’s specific procedure, initially using a servo tester during the preliminary testing phase (Figure 8b), and later via software through the aircraft’s FC. During the calibration process, sound sequences provide feedback, indicating the status and progress of the ESC calibration.

The third optional part of the experiment involves experimentally determining the Kv (velocity constant) of the BLDC motor using measurement equipment. During this phase, the motor is mounted on the setup along with the connected ESC. After becoming familiar with the experimental setup software, an automated script is executed to measure the motor’s velocity constant. For safety reasons, it is crucial to emphasize once again that all three parts of this hands-on experiment must be conducted without the propeller.

**Figure 9:**
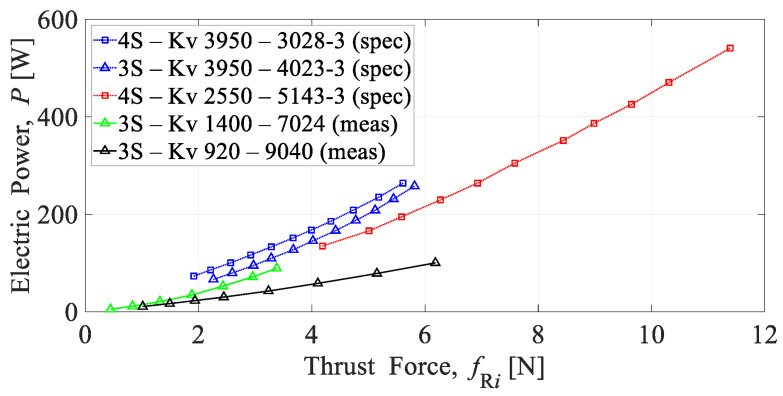
Characteristics of considered electric propulsion unit setups.

Regarding the size of the engineering education platform, it utilizes small BLDC motors with velocity constants Kv > 1000, in combination with propellers ranging from 2 to 10 inches, and batteries consisting of 3 to 4 cells. It is important to note that this type of UAV is a high-energy consumption system, considering that rotary wings must counteract gravity force. To power all the mentioned modules, an energy module typically consists of one or more LiPo batteries, along with all the necessary wiring and connectors for other subsystems. The overall performance depends on the battery’s capacity and mass. Recommended propulsion setups are given by the manufacturer in the form of tabular specifications, from which characteristics can be obtained as shown in Figure 9.

Experimental testing of such propulsion units can be incorporated into the educational process. To understand the principles of operation of electronic and electromechanical converters, several steps can be considered. In addition to the actual implementation of the experiment using equipment and sensors for measuring mechanical and electrical quantities, additional steps include data acquisition and the visualization of measurement data. Based on the experimental data, characterization can be performed, which is crucial in engineering education, as it links the hardware with the mathematical description of the multirotor aircraft. In Figure 9, along with the characteristics provided by the propulsion manufacturer, the characteristics derived from the conducted experimental tests are also shown as an example.

### 4.2. Design and Prototyping of Multirotor Airframe

The multirotor educational platform serves as an excellent tool for hands-on learning, directly linking to several learning outcomes in the field of design, prototyping, and manufacturing of mechatronic systems. Through engagement with this platform, students can gain practical experience in identifying and selecting appropriate materials and processing procedures essential for the production of complex mechatronic systems (IUP11). The process of constructing airframe parts and multirotor assemblies strengthens their understanding of the principles of strength and deformation, as well as kinematics and dynamics, providing a solid foundation in mechanical design (IUP13). Furthermore, creating 2D technical documentation and developing 3D models for a multirotor platform can improve students’ expertise in modern engineering design, tools, and techniques, preparing them for real-world applications in mechatronic systems development (IUP17). Two case studies will be presented in which off-the-shelf components were selected and tested, and the design and manufacturing process of the airframe parts are demonstrated. When producing adaptable educational UAVs, several features are important to consider. First, safety should be prioritized, given the characteristics of this type of UAV and its intended extensive use in laboratory exercises. Additionally, rapid prototyping (RP) technologies are convenient, as they speed up the manufacturing process and enable in-house replacement, modification, and improvements of airframe parts. The next objective in the design phase is to achieve easy integration with setups involving independent testing for individual DOFs or groups of DOFs during different testing phases.

**Figure 10:**
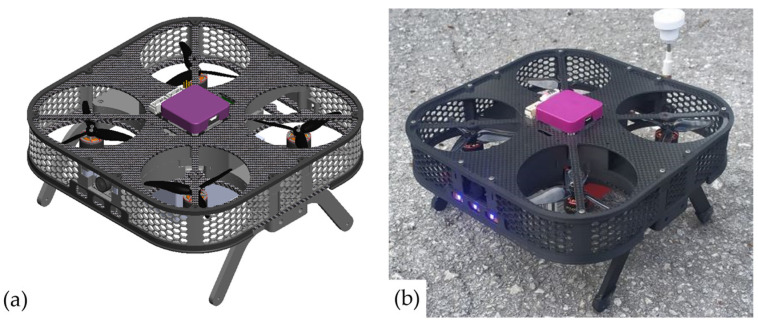
Experimental educational quadrotor: (**a**) 3D model assembly; (**b**) Ready to fly prototype.

Consistent with the learning outcome of IUP11, it is crucial to use lightweight materials for multirotor UAVs, as is the case for all aircraft types. Airframe parts are produced from composite materials, plastic, and light alloys (such as aluminum). Several design approaches exist for multirotor UAVs based on size and power requirements. Medium-sized multirotors, capable of carrying payloads around 1 kg and heavier models, typically employ composite materials consisting of tubes and sheets, along with plastic airframe components. Smaller aircraft follow similar principles, where composite parts can be replaced with 3D-printed plastic airframe parts. Furthermore, the design of multirotors as educational platforms will be showcased, incorporating two rapid prototyping technologies. Figure 10a shows a 3D model of the experimental educational quadrotor assembly. The prototype consists of a T-Motor F1408 BLDC motors with velocity constant Kv = 3950 on which HQprop T3X2.5X3 propellers are mounted. Additional features of this assembly include a plug-in battery and a simple replacement of the Cube FC series (purple, black, red). Figure 10b shows a ready-to-fly quadrotor prototype that will be used in engineering education, especially for indoor tests. The production process for manufacturing airframe parts from composite (carbon fiber) sheets is straightforward. The required machines are relatively inexpensive and readily available, allowing this production technology to be integrated into the engineering education process. First, the two-dimensional geometry of the part is saved in .dxf format, compatible with CNC router software. Next, tools and cutting parameters are selected, and the g-code is generated. A 3-axis CNC router with a milling motor is then used to produce the airframe parts from carbon fiber sheets of various thicknesses. This production technology enables the simple production of parts from composite materials, which are mechanically stressed.

Multirotors offer an engineering perspective on airframe construction that can be related to the following learning outcome: to construct assemblies in accordance with the laws of strength and deformation of materials, kinematics, and dynamics (IUP13). Given that multirotor UAVs are often used in applications involving small series or prototypes, it is important to consider rapid prototyping technologies during the design phase. Regarding this learning outcome, AM technologies will be considered for manufacturing designed airframe parts of educational platforms. Additionally, the design of a multirotor platform is a challenging and interesting case study from the perspective of engineering education in the field of AM technologies.

Regardless of AM technology, parts are built layer by layer. For example, selective laser sintering (SLS) technology uses powder materials sintered by laser-generated thermal energy, while fused deposition modeling (FDM) technology melts solid polymer materials into a semi-liquid state that is extruded through a nozzle to form parts. Due to the cost of equipment and materials, FDM technology is suitable for integration into the engineering education process, particularly for conducting hands-on exercises.

**Figure 11:**
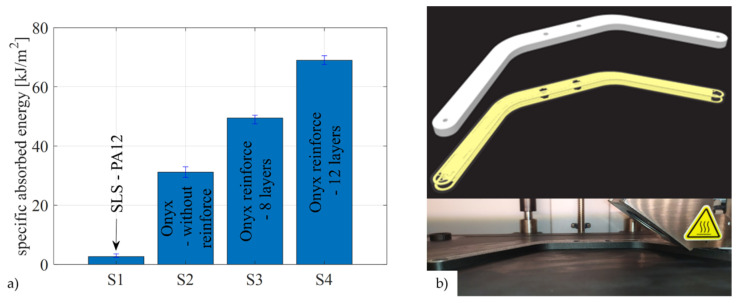
Additive manufacturing of parts: (**a**) Material characterization; (**b**) Printing parameters for landing gear.

For components subjected to lower mechanical loads, FDM technology using the Prusa i3 MK3s 3D printer (Prusa Research a.s., Prague, Czech Republic) and PrusaSlicer software (version 2.4.0) are utilized. PETG material is chosen due to its greater resistance to UV rays. The mechanical properties of the anisotropic airframe part are influenced by the selected material, 3D printing parameters (such as layer thickness, number of outer layers, and infill type and percentage), and the part’s geometry. For airframe components that require higher mechanical properties, it is important to consider other AM technologies in the design process. The process of manufacturing parts using continuous fiber fabrication (CFF) technology is planned to be integrated into the education process. CFF technology is essentially an extension of FDM technology that uses direct reinforcement and requires two nozzles. The first nozzle deposits the matrix material, while the second nozzle adds the reinforcing fibers, creating a composite sandwich structure. For airframe parts subjected to higher mechanical loads, CFF technology is employed using the Markforged Onyx Pro 3D printer (Markforged, Waltham, MA, USA) and Eiger slicer. Special attention is given to the production of airframe parts for the propulsion configuration and landing gear. The goal is to incorporate both FDM and CFF technologies into engineering education, enabling students to gain hands-on experience with the associated hardware and software tools. Figure 11a displays the material characteristics in a Charpy impact test [45], while Figure 11b shows the landing gear part during the adjustment of the CFF 3D printing parameters, and printing execution.

**Figure 12:**
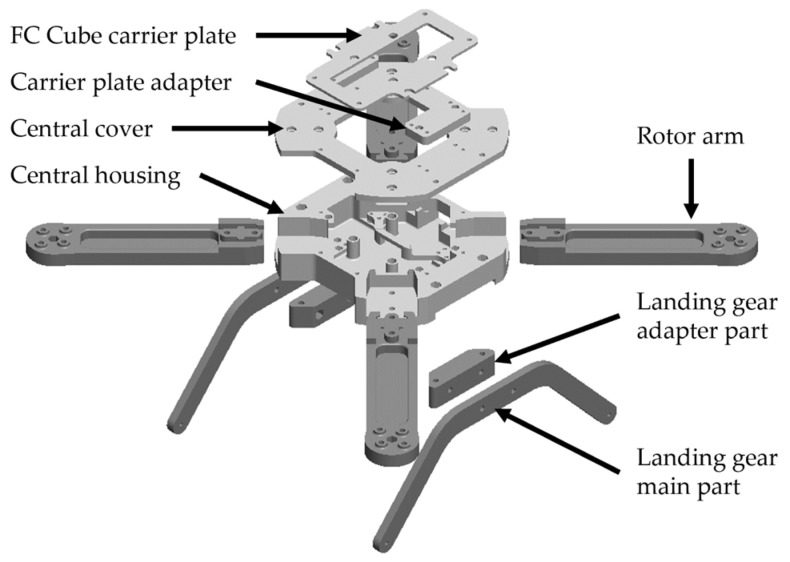
Experimental educational quadrotor—3D model airframe assembly.

The design process for airframe parts and the assembly of UAVs culminates in achieving the following learning outcome related to the hardware aspects of the aircraft: creating 2D technical documentation and constructing a 3D model of mechatronic systems (IUP17). Various Computer-aided design (CAD) software packages can be used for modeling airframe parts, with the following sections focusing specifically on the SOLIDWORKS (2021) and CATIA (V5) software packages (Dassault Systèmes SE, Vélizy-Villacoublay, France). The aforementioned software packages are used because the Karlovac University of Applied Sciences has educational licenses and their implementation in the education process is possible. In addition to 3D modeling and the creation of 2D documentation within the software, it is also possible to perform simulations regarding mechanical loads. Multirotor propulsion configurations are particularly interesting for parametric modeling, further highlighting this case study as highly applicable in the engineering education process. Figure 12 shows the 3D model of the airframe construction assembly. In the figure, parts made using FDM technology with PETG material are depicted in light gray, while parts produced with CFF technology, using an Onyx carbon-reinforced nylon matrix and fiberglass reinforcement, are depicted in dark gray. As an additional hardware contribution to this paper, the presented educational aircraft prototype is available as an open-source 3D airframe model on the Thingiverse online repository [46]. The proposed airframe construction is very cost-effective compared to other components of the educational platform.

### 4.3. Hands-On Training Through Experimental Hardware Testing

**Figure 13:**
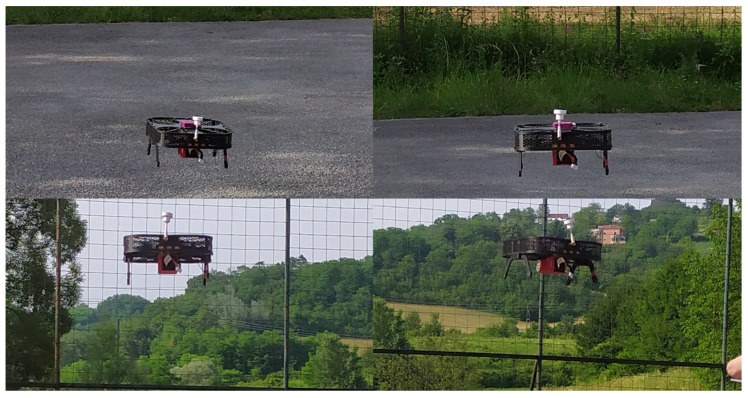
Preliminary outdoor experimental testing.

To effectively automate processes in engineering systems, preliminary tasks include connection and integration of various components, such as sensors, actuators, control components, microcomputers, and accompanying equipment. This approach directly aligns with the learning outcome of IUP21. In this context, the multirotor platform once again proves to be an excellent tool for integration into engineering education. For students to become proficient with a multirotor platform, it is essential to learn to pilot such an aircraft in a safe environment. Before the first flight, it is mandatory to perform initial calibrations of the UAV system. The propulsion system should be calibrated first, followed by the control system, which includes various sensors within the inertial measurement unit (IMU). Additionally, the airframe reference and controller parameters must be appropriately adjusted, failsafe mechanisms set, and configurations for additional modules such as RC, GNSS, and telemetry completed. During hands-on training, students are using Mission Planner (1.3.82) and QGroundControl (4.4.3) software for pre-flight preparations, flight operations, and flight analysis. Figure 13 shows the example of outdoor testing of the quadrotor within a secure enclosure and under potential wind impact.

**Figure 14:**
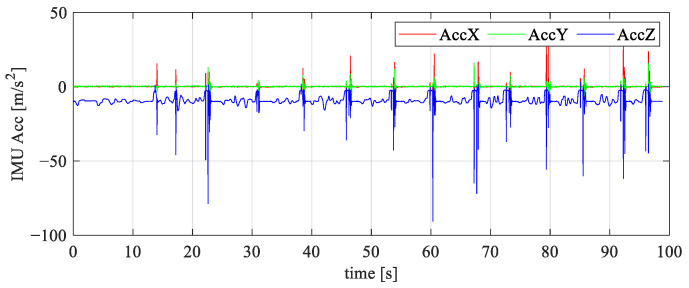
Accelerometer readings from the FC recorded during rough landing test.

It is important to emphasize a few considerations related to outdoor flight. While testing can be performed independently inside facilities, permits from regulatory bodies are mandatory for any flights performed outside a facility. This requirement is less ideal from the education perspective, especially since system failures can occur during experiments. As these are prototypes, the airframe parts are also subject to testing. This is particularly engaging for students, as it allows them to design, prototype, and test their own projects. Figure 14 illustrates accelerometer responses during rough landing tests of the educational quadrotor platforms. This is crucial because the aircraft is intended for student training, where they pilot such systems for the first time. The absence of damage on the landing gear parts after extensive testing indicates the successful parameter selection during the design and production stage. However, most of the planned hands-on training will be performed indoors, in a secure environment, or with security elements included. Furthermore, the paper will describe indoor experiments focusing on software aspects aligned with specific learning outcomes.

## 5. Multirotor UAV Software Aspects

The multirotor educational platform provides a comprehensive hands-on experience that aligns with several learning outcomes related to software and control aspects of engineering education. Through the integration of flight controllers and various sensors, students can design and program software for multirotors, gaining practical skills in embedded systems. This aligns with the design of electronic devices with microcomputers and the creation of software solutions (IUP16). This paper emphasizes two learning outcomes that are strongly related to multirotor platforms, presenting the main elements of hands-on engineering education. Firstly, students learn to calculate control algorithm parameters (IUP18) which are essential for stabilizing and maneuvering the aircraft, offering insight into the real-time control of dynamic systems. The next outcome, which is closely related to the multirotor platform, allows students to program, analyze, simulate, and demonstrate the operation of multirotors, as well as to plan and adjust flight trajectories, analogous to robotic manipulator trajectory planning (IUP23). Finally, IUP25 is addressed by integrating computers with software to collect, measure, and display flight data, providing a holistic approach to data-driven analysis and system optimization. This multifaceted educational experience ensures that students are well-prepared for the various technical challenges in the fields of mechatronics and aerospace engineering.

### 5.1. PX4 Ecosystem

The control module hardware of the educational multirotor platform is based on the Cube FC using the Dronecode (PX4 v1.13.3) autopilot. A key feature of that ecosystem from, an education standpoint, is its open-source software architecture and compatibility with various modules, such as the GNSS, telemetry, and radio receiver. The primary advantage of this ecosystem is its integration with the MATLAB Simulink (R2021b) software package via the UAV Toolbox Support Package, which accelerates training for both software and hardware solutions. Educationally, this enables the integration of learning outcomes IUP15 and IUP23. The presented hardware solutions are integrated with the MATLAB ecosystem to facilitate experiments on various aspects of the multirotor UAV. This section outlines the main elements of the custom experimental firmware.

From a control design perspective, the multirotor platform is a second-order multivariable system that is inherently unstable and highly nonlinear, making it a challenging control object. Using custom firmware as a standardized control scheme can address several training cases, including simulations, software-in-the-loop testing, hardware-in-the-loop testing, and flight testing. Regarding the reference state, there are two main scenarios for flight settings. For autonomous flight, it is necessary to plan a mission where the UAV follows a predefined reference trajectory, assuming that the state of the aircraft (for all six DOFs) is known in real time. In the case of remote control, the pilot directly controls the UAV’s movement by setting the reference state. Remote control can manage the aircraft’s position and orientation, with additional features such as arming the aircraft, changing flight modes, and adjusting the controller parameters. Telemetry components are used for real-time system monitoring, providing two-way communication. This allows not only for receiving data on the aircraft state, energy consumption, and other metrics but also for adjusting system parameters from the base station. The entire multirotor UAV system’s status is logged on the SD card in the FC, which is crucial for analyzing flight details.

**Figure 15:**
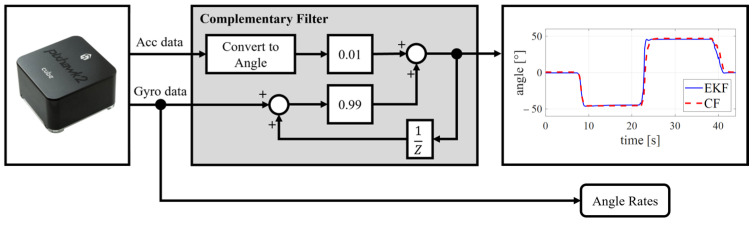
Schematic representation of a complementary filter (CF).

As an introductory experiment integrated as a laboratory exercise, the Cube FC will be used to familiarize students with the PX4 ecosystem within the MATLAB software package. Given its standard use in education, the availability of extensive educational materials, and its integration potential across various courses in the mechatronics engineering curriculum, this experiment will demonstrate the use of a complementary filter (CF), as schematically illustrated in Figure 15. The experiment involves a set of sensors contained within the inertial measurement unit (IMU) integrated into the FC. The IMU includes an accelerometer, gyroscope, magnetometer, and barometer. The Cube FC hardware offers dual or triple redundancy of integrated sensors, depending on the Cube series. These data is crucial for determining the orientation and altitude of the multirotor UAV. Through this hands-on exercise, students enhance their skills in areas such as signal processing and filtering, state estimation, sensor fusion, and more.

**Figure 16:**
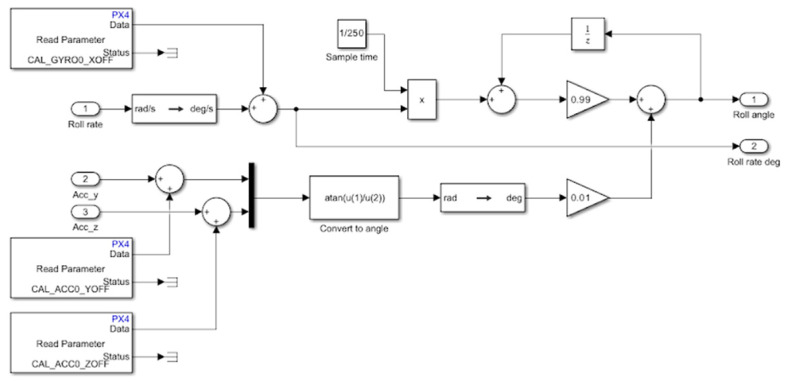
Implementation of a complementary filter for one DOF in MATLAB Simulink (R2021b).

The open architecture, compatibility with MATLAB, and modular design of the proposed firmware enable the implementation of custom filters and estimators tailored for educational purposes. A complementary filter (CF) is associated with the sensors block that represents the control feedback link. Implementing and testing a CF for estimating the orientation of the multirotor UAV is straightforward and educationally beneficial. To determine the multirotor’s orientation (attitude), a CF uses data from the accelerometer and gyroscope. In addition to the CF, built-in estimators such as the Extended Kalman Filter (EKF) and other software solutions can be employed for educational purposes, depending on the level of hands-on training. Figure 16 illustrates the MATLAB Simulink model of a complementary filter for one DOF.

### 5.2. Control Design Experiment Series

A series of experiments related to control design is strongly aligned with the learning outcome of calculating the parameters of control algorithms for various technical processes (IUP18). These experiments will guide students through the principles and practices of control system design, using a multirotor UAV platform as a practical example. Hands-on training includes designing and implementing control algorithms, adjusting parameters to achieve the desired performance, and analyzing flight data. This experiential approach ensures that students not only understand theoretical concepts but also develop practical skills in the design and application of control systems. Experimental setups that allow the testing of individual DOFs and their combinations provide several key advantages. First, starting with a 1DOF system enables students to grasp fundamental control concepts in a simplified context. The effects of changes in control parameters on system dynamics foster a clear understanding of the relationship between theoretical control design and practical results. As students progress to 2DOF and more complex control scenarios, they face the additional challenges of managing interactions between multiple axes and coordinating control actions to achieve stable flight.

The proposed approach, which focuses on varying DOFs, helps students gradually build confidence and competence. Testing these setups provides valuable insights into the real-world performance of control algorithms under different conditions. Furthermore, comparing these experiments with classical control experiments, such as “ball and beam” or “ball and plate” [47] systems, emphasizes their educational value. Unlike these simpler educational settings, the multirotor UAV platform introduces students to more complex and realistic control problems. By progressing from 1DOF to 3DOF attitude control and towards position control, students incrementally apply and refine their control algorithms while encountering and solving practical challenges similar to those in advanced engineering fields. This hands-on experience is crucial for understanding the complexities of multivariable control systems and prepares students for advanced applications in robotics, aerospace, and other fields requiring dynamic system control. This practical exposure to control design and testing significantly enhances their abilities, meeting the educational objectives of the learning outcome.

**Figure 17:**
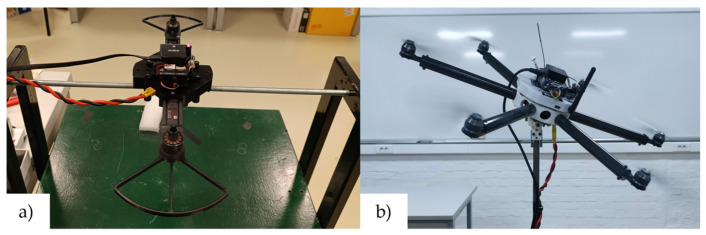
Multirotor experimental indoor setups: (**a**) 1DOF testing; (**b**) 3DOF testing.

The proposed sequential experiments, with a focus on varying DOFs, can be easily implemented as hands-on training in both classroom and laboratory environments, allowing students to gain practical experience and reinforce theoretical concepts through direct application. The first experiment focuses on controlling a single DOF (Rotation Angle), where a shortened configuration with two rotors or a complete aircraft configuration can be used in combination with a one-axis experimental setup. The second experiment involves controlling 2 DOFs (roll and pitch angles). In the subsequent Attitude Control experiment, students design control across all rotational axes (roll, pitch, and yaw). For those two experiments, an experimental setup consisting of a three-axis gimbal joint is used, which allows one axis to be locked [48]. The fourth experiment centers on position control using remote control elements to simulate realistic flight scenarios. Position control will be discussed in greater detail in the next subsection concerning the subsequent learning outcome. Figure 17 shows the tests of the multirotor platform for the proposed series of experiments.

A particular experiment consists of several key steps: designing the controller, implementing and testing the algorithm on a multirotor configuration, adjusting and calculating the controller parameters, and analyzing the flight log. The control design step relies on conventional control techniques covered in control theory courses. For educational purposes, the Linear Quadratic Regulator (LQR) and the cascade Proportional Integral Derivative (PID) control algorithms were chosen. To implement the algorithm and test it on the aircraft, it is necessary to define the multirotor configuration parameters. These parameters describe a signals allocation where the inputs are control variables, and the outputs are control signals for the electric propulsion configuration. Conventional multirotor configurations, such as quadrotors, hexarotors, or octorotors with planar geometric arrangements, have four independent control variables. As previously mentioned, conventional configurations are underactuated systems with strongly coupled dynamics. Multirotors are a compelling platform as they represent typical aerial robots. By selecting different configuration parameters, it is possible to increase the number of control variables, which subsequently alters the controller design. Unconventional aircraft configurations introduce different requirements for flight planning, further enriching the educational experience.

**Figure 18:**
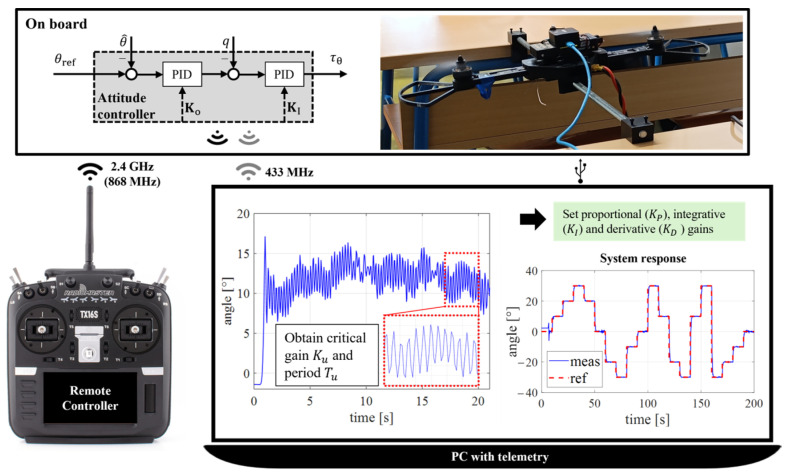
Schematic representation of PID tuning based on the Ziegler-Nichols method.

From an educational standpoint, it is important to highlight the risks associated with testing microcontroller program solutions for multirotors without properly setting the control system parameters. This step is crucial for ensuring safe and desired flight performance. One of the experiment steps shown involves adjusting the parameters of the internal control loop of a cascade PID controller for a one DOF experimental setup. This experiment demonstrates the Ziegler-Nichols method for tuning the angle rate parameters of the inner PID control loop. This method allows for quick and easy parameter adjustments through the defined sequence. In Figure 18, the step of adjusting and calculating the parameters of the control algorithm is shown schematically.

Since UAVs are predominantly used for outdoor operations, it is crucial to account for external disturbances affecting the system, such as wind gusts. Familiarizing students with these aspects of UAV systems is essential, as they are critical for practical applications. To address these challenges, numerous control techniques have been developed to overcome the limitations of conventional methods. The application of conventional disturbance observers is often constrained by the need to know the mathematical model of the controlled system. This paper refers to Active Disturbance Rejection Control (ADRC) techniques based on the Extended State Observer (ESO), discussed in previous research, such as [49,50]. Enhancing the performance of the ADRC methodology remains an active area of research, particularly in UAV control applications [51,52]. Building on prior research [31], courses such as Fundamentals of Automatic Control and Robotics will include demonstrations of Hardware-in-the-Loop (HIL) simulations. These simulations will compare the performance of conventional control techniques with robust control methods [53], providing students with practical insights into these advanced systems.

### 5.3. Framework for Position Control

Through the integration of flight controllers and various sensors, students can program, analyze, simulate, and demonstrate the control, state estimation and trajectory planning of aerial robots (IUP23). Experimental tests can be categorized into outdoor and indoor tests based on educational purposes, with the choice of sensor packages for positioning the aircraft in 3D space depending on the testing environment. For outdoor tests, it is crucial to ensure a secure environment, such as a safety cage. Positioning can be achieved using the GNSS system, with the Real-time kinematic (RTK) [54] module providing more precise positioning. Additional sensors such as the IR Lock sensor for accurate landing and the optic flow sensor, can be used in both outdoor and indoor settings. Indoor experiments will primarily be integrated into the educational system, continuing the series of hands-on experiments.

**Figure 19:**
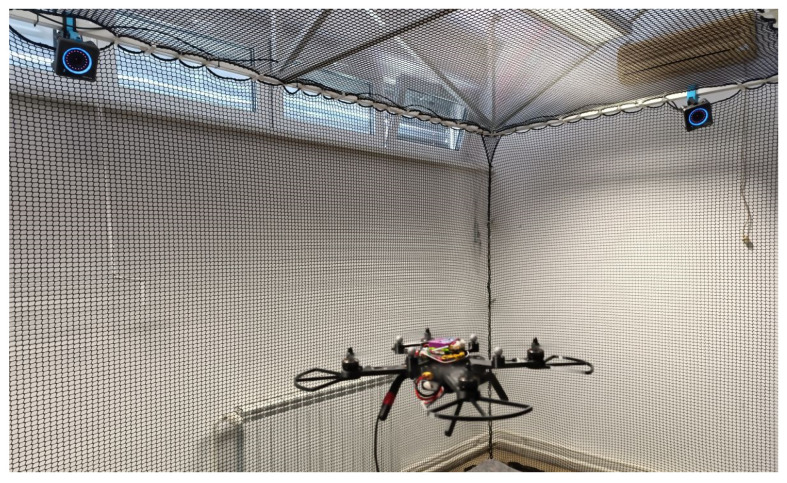
Experimental testing of the quadrotor position control using the Optitrack system.

A series of experiments was conducted with the primary goal of verifying the motor mixer for various multirotor UAV configurations. These configurations were tested using motion capture systems, which serve as external sensors providing precise position and orientation data, enabling the simultaneous tracking of multiple objects. Such systems are extensively used across different fields and are valuable assets in modern research and educational laboratories. For hands-on education, a setup with six Optitrack Primex cameras (NaturalPoint, Inc., Corvallis, OR, USA) [55] will be utilized. Figure 19 illustrates the testing of the quadrotor platform using the Optitrack system, with markers mounted on the airframe. A custom experimental framework was utilized, with the source code available on GitHub [56,57]. This approach directly supports another learning outcome: integrating a computer with software support for data collection, measurement, and display on a computer (IUP25).

## 6. Discussion

This paper explores the application of multirotor UAVs in engineering education, focusing on their integration across mathematical modeling, software, and hardware aspects. It outlines the relevant learning outcomes and demonstrates how these aspects can collectively address various educational objectives. This approach facilitates a comprehensive understanding of mechatronic systems, bridging theoretical concepts with practical hardware and software applications. The paper provides an in-depth presentation of how these three aspects are interconnected within the undergraduate professional study curriculum.

Given that the undergraduate professional study of mechatronics in Karlovac is currently undergoing revision and adjustment to the Croatian Qualifications Framework, and given that the procurement process for components to create educational prototypes is still ongoing, the use of UAVs is not yet fully implemented. Currently, multirotor UAVs are employed as educational platforms using computer equipment and demonstration models. Table 3 lists the courses in which UAVs are currently used for the purpose of engineering education.

Regarding the Actuators and Mechanisms course, UAVs are utilized in lectures to teach the performance characteristics of electric propulsion units that constitute the propulsion systems of multirotor UAVs. Subsequently, experimental measurements and data acquisition are conducted during laboratory exercises. Students’ work involves preparing a report that presents the characteristics of the assigned propulsion system, which is then evaluated.

**Figure 20:**
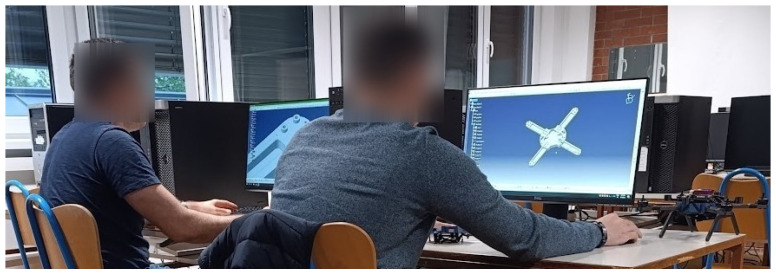
Three-dimensional modeling of parts and assembly of multirotor aircraft as part of classes at Karlovac University of Applied Sciences.

The Design of Mechatronic Systems course generally consists of lectures and design exercises. Students work independently on a project assignment that includes the phases of sketch design, part design, assembly, and the technical documentation of the platform. Students use the CATIA (V5) software package to design a multirotor UAV, as shown in Figure 20. Given the versatility of multirotor UAVs, the project tasks can be individualized by specifying different platform configuration parameters. From a practical standpoint, since the software is installed on computers in a lab with a busy teaching schedule, students face limited time resources for completing their project assignments. This challenge could be addressed by utilizing free software such as Onshape (Onshape Inc., Boston, MA, USA) [58] or FreeCAD [59].

**Figure 21:**
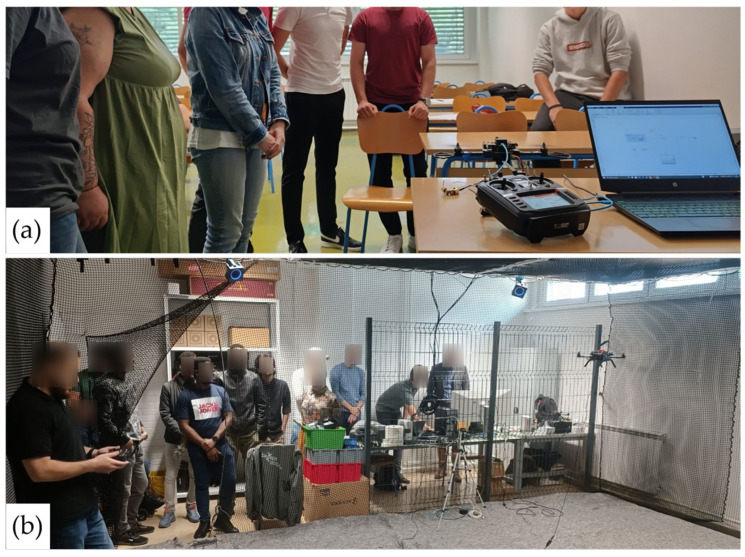
Workshops related to software aspects of multirotor UAV: (**a**) 1DOF attitude control; (**b**) Position control using the Optitrack system.

Regarding the remaining two courses, UAVs have already been integrated into part of the teaching. For the laboratory component, workshops using prototypes were conducted to address software aspects, as shown in Figure 21. With the procurement of components and equipment, a set of multirotor platforms will be produced for use in robotics and automation courses. These platforms will provide students with practical, hands-on experience, effectively bridging the gap between theoretical knowledge and real-world application in robotics and automation. By working with these platforms, students will apply classroom concepts to real-world scenarios and gain practical skills highly valued in the industry.

With regard to the Fundamentals of Automatic Control course, the last section involves the synthesis of the cascade control algorithm for the multirotor UAV. Future laboratory exercises will involve the implementation of control algorithms and the tuning of controller parameters for experimental setups with a single DOF. It is important to note that, for student safety, these setups must include fully enclosed propellers. This precaution is necessary because the setups will be operated in close proximity to students in the classroom or laboratory during experiments.

Since only partial feedback was received from students after conducting the workshops, a potential challenge lies in preparing instructions for laboratory exercises that students complete individually. If the instructions are too simple, students may not actively engage in learning during the exercises. On the other hand, if the instructions lack sufficient detail, students may struggle to complete the exercises independently without the lecturer’s assistance. It is essential to find the right balance, and the instructions will be updated as needed after the end of the academic year.

In the Robotics course, students engage in lectures covering model derivation and implementation. They also participate in a demonstration session where the fundamental concepts of multirotor aircraft operation are presented in a laboratory setting. Additionally, plans are in place to implement laboratory exercises focusing on position control and motion planning using the Optitrack system.

## 7. Conclusions

This paper presents a systematic approach to describing multirotor aircraft, positioning it as an ideal platform for engineering education. It covers the mathematical modeling of the aircraft and the configuration of the propulsion module. From a hardware perspective, the focus is on the design of airframe components for educational platforms. These developed open-source, 3D-printed multirotor platforms are expected to enhance hands-on learning. On the software side, the emphasis is placed on control design, though the solution is flexible enough to explore other areas. The paper introduces a series of control design experiments using the multirotor platform, featuring experimental setups that allow testing of individual DOFs and their combinations. Through this platform, students can apply theoretical concepts to real-world scenarios, acquiring practical skills highly valued in the industry. The proposed hardware and software solutions are intended for integration into the mechatronics programs at Karlovac University of Applied Sciences and the Zagreb University of Applied Sciences.

## Figures and Tables

**Table 1:** Learning outcomes of the undergraduate professional mechatronics study program according to HKO [40].

Learning Outcome	Learning Outcomes of the Undergraduate Professional Study of Mechatronics
IUP1	Apply mathematical and physical laws to engineering problems.
IUP2	Use a foreign language in professional literature and everyday and professional communication.
IUP3	Apply the principles of business communication within the profession: recognize the needs of clients, and present information, ideas, problems, and solutions to professional and non-professional audiences.
IUP4	To acquire new knowledge, skills, abilities, and responsibilities.
IUP5	Adapt to work in project teams.
IUP6	Identify, model, and solve engineering problems.
IUP7	Align engineering activities with the needs of product and service users.
IUP8	Be flexible and adapt in finding technical solutions while respecting basic ethical principles, legal norms, and rules of the profession.
IUP9	Use techniques, skills, and modern tools necessary for engineering practice
IUP10	Critically evaluate professional facts, terms, procedures, principles, and theories in the field of mechatronics.
IUP11	Recognize and propose adequate types of materials and machining procedures for the production of mechatronic systems.
IUP12	Dimensioning and selecting standardized elements of precision mechanics, machine elements, and assemblies in the process of manufacturing various mechanisms.
IUP13	Construct machine elements, assemblies, and mechanisms following the laws of strength, deformation, kinematics, and dynamics.
IUP14	To know the principles of operation of electronic and electromechanical converters.
IUP15	Analyze the behavior of mechatronic systems by modeling and simulating.
IUP16	Design electronic devices with microcomputers and create microcomputer software solutions.
IUP17	Create 2D technical documentation and construct a 3D model of mechatronic systems.
IUP18	Calculate the parameters of the control algorithm for the control of various technical processes.
IUP19	Automate systems using pneumatic and hydraulic elements.
IUP20	Program programmable logic controllers using current platforms and programming languages.
IUP21	Select and connect sensors, actuators, microcomputers, programmable logic controllers, and accompanying equipment for the automation of production processes.
IUP22	Know the principles of the quality assurance system in mechatronics.
IUP23	Program, analyze, simulate, and demonstrate the operation of robots and plan the trajectories of robot manipulators.
IUP24	Maintain mechatronic systems.
IUP25	Integrate the computer with software support in the process of data collection, measurement, and data display on the computer.
IUP26	Identify and solve problems in the production process related to mechatronic elements, circuits, or devices.
IUP27	Plan, monitor, and control the production of mechatronic system elements.

**Table 2:** Overview of components and modules for educational UAV operation.

Components and Modules	Attitude Control (1–3 DOF)	Remote Control	Position Control	
			Outdoor	Indoor
Airframe parts	✓	✓	✓	✓
Propulsion components	✓	✓	✓	✓
Battery	✓	✓	✓	✓
FC (carrier board + telemetry + battery sensor)	✓	✓	✓	✓
Remote control (Transmitter + receiver)	(✓)	✓	(✓)	(✓)
GNSS module			✓	
Companion computer + Optitrack system				✓

**Table 3:** Courses considered for UAV use in the undergraduate professional study of Mechatronics.

Course Name	Total ECTS	UAV Use ECTS	Teaching Activity	Related Study Learning Outcomes	Duration (Weeks)
Fundamentals of Automatic Control	5	1	Lectures, Laboratory Exercises	IUP15, IUP18	3
Robotics	5	1.5	Lectures, Laboratory Exercises	IUP1, IUP6, IUP23	4
Actuators and Mechanisms	5	1	Lectures, Laboratory Exercises	IUP14, IUP25	2
Design of Mechatronic Systems	5	1	Lectures, Project Assignment	IUP13, IUP17	3

## Data Availability

The original data presented in the study are openly available in [Thingiverse] at [https://www.thingiverse.com/thing:6866923].
